# Current and Emerging Therapies for Limbal Stem Cell Deficiency

**DOI:** 10.1093/stcltm/szab028

**Published:** 2022-03-18

**Authors:** Abdelrahman M Elhusseiny, Mohammad Soleimani, Taher K Eleiwa, Reem H ElSheikh, Charles R Frank, Morteza Naderan, Ghasem Yazdanpanah, Mark I Rosenblatt, Ali R Djalilian

**Affiliations:** 1 Department of Ophthalmology and Visual Sciences, University of Illinois at Chicago, Chicago, IL, USA; 2 Department of Ophthalmology, Harvey and Bernice Jones Eye Institute, University of Arkansas for Medical Sciences, Little Rock, AR, USA; 3 Department of Ophthalmology, Farabi Eye Hospital, Tehran University of Medical Sciences, Tehran, Iran; 4 Department of Ophthalmology, Faculty of Medicine, Benha University, Benha, Egypt

**Keywords:** corneo-scleral limbus, limbal stem cell niche, limbal epithelial stem cell deficiency, extracellular matrix

## Abstract

The corneal epithelium serves to protect the underlying cornea from the external environment and is essential for corneal transparency and optimal visual function. Regeneration of this epithelium is dependent on a population of stem cells residing in the basal layer of the limbus, the junction between the cornea and the sclera. The limbus provides the limbal epithelial stem cells (LESCs) with an optimal microenvironment, the limbal niche, which strictly regulates their proliferation and differentiation. Disturbances to the LESCs and/or their niche can lead to the pathologic condition known as limbal stem cell deficiency (LSCD) whereby the corneal epithelium is not generated effectively. This has deleterious effects on the corneal and visual function, due to impaired healing and secondary corneal opacification. In this concise review, we summarize the characteristics of LESCs and their niche, and present the current and future perspectives in the management of LSCD with an emphasis on restoring the function of the limbal niche.

## Graphical Abstract

**Graphical Abstract:**
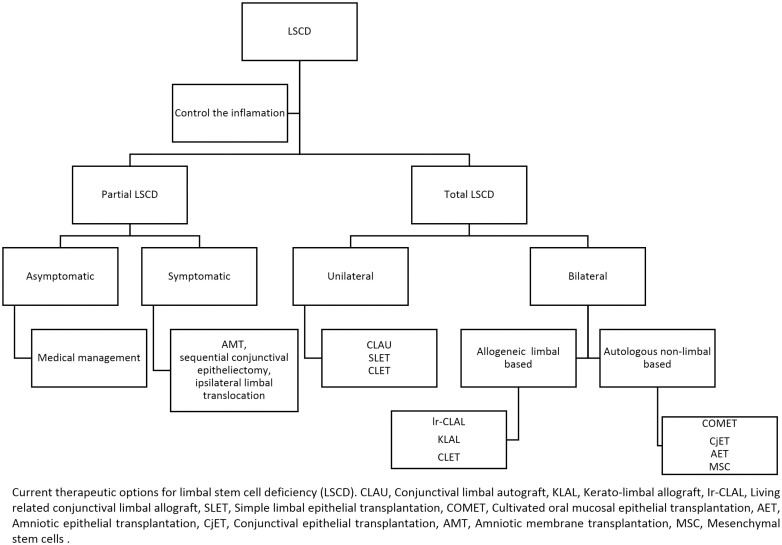

**Significance Statement**

In this concise review, we summarize the characteristics of limbal epithelial stem cells and their niche, and present the current and future perspectives in the management of limbal stem cell deficiency with an emphasis on restoring the function of the limbal niche.

## Introduction

Limbal epithelial stem cells (LESCs) are unipotent adult stem cells that reside in an anatomically distinct stem cell niche within the limbus. The limbal niche is a specialized microenvironment with unique physical, autocrine, paracrine, and multicellular properties critical to the maintenance of healthy LESCs.^1,2^ Lying deeply in the basal epithelial layer of a healthy functioning limbal niche, LESCs are responsible for lifelong regeneration of mature corneal epithelium. Limbal niche dysfunction provoked by any significant corneal pathology can perturb the LESCs and predispose to limbal stem cell deficiency (LSCD).^3^

Herein, we review the structure and function of the limbal niche, the associated pathologies, and the therapeutic options for LSCD.

## Limbal Niche

**Figure 1.**
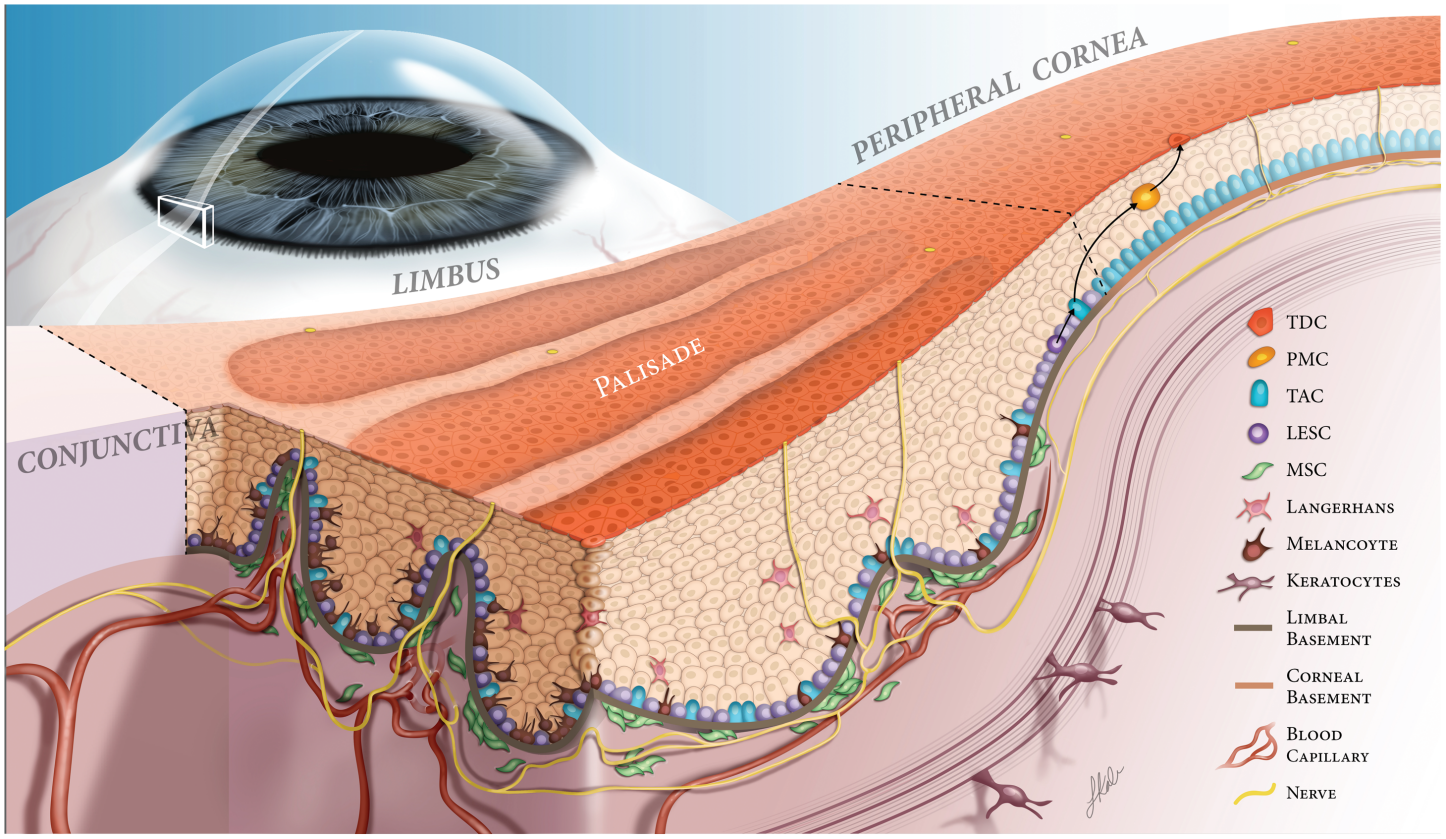
Limbal niche. Illustration of the limbal niche, focusing on the Palisade of Vogt. The Palisades of Vogt form crypts in the limbal epithelium, allowing for close contact of LESCs to supportive cells including melanocytes, keratocytes, mesenchymal stem cells and Langerhans cells. These cells, along with the basement membrane and neurovasculature, provide growth factors, nutrients, and structural support to promote proper LESC proliferation and differentiation. At the border of the limbal and corneal basement membranes, LESCs divide into progenitor cells, or transient amplifying cells (TAC). The TACs divide into postmitotic cells (PMCs) and migrate centrally. These PMCs differentiate into terminally differentiated epithelial cells (TDCs) to replace lost cells on the corneal surface. Illustration with permission from Yazdanpanah G, Haq Z, Kang K, Jabbehdari S, l Rosenblatt M, Djalilian AR. Strategies for reconstructing the limbal stem cell niche. *Ocul Surf.* 2019;17(2):230-240.^2^

The limbal niche is a multicellular microenvironment with a unique extracellular matrix (ECM) and various signaling molecules that supports the function of the LESCs.^4-6^ The classic limbal architecture is comprised of the palisades of Vogt, which are undulations of the stroma and epithelium, analogous to the rete ridges in the skin (Fig. 1).^5,7^ They are more prevalent in the superior and inferior limbus and less notable in the nasal and temporal limbus.^8^ The LESCs are located at the basal layer of the limbal epithelial undulations. There are also unique clusters of CD90 and CD105 positive mesenchymal stem/stromal cells (MSCs) found adjacent to the basal epithelium.^2,9^ Cellular and molecular analysis has unveiled distinctive gene expression and ECM protein profiles that are mandatory for the maintenance of limbal niche homeostasis.^5^ A physical crosstalk between limbal MSCs and LESCs was based on MSCs projections that were found to penetrate the basement membrane and have direct contact with LESCs.^10^ Additionally, MSCs secrete factors that support and maintain LESCs clonal proliferation and differentiation via various signaling pathways.^3^ The ECM of the limbus is distinct from corneal stroma in that it is enriched with vitronectin, fibronectin, α2 and β2-laminin, Tenascin C, α1,α2, α5, and α6 collagen type IV collagen and Wnt ligands that are specifically crucial to the maintenance and function of LESCs.^6,11,12^ Limbal niche provides a soft environment for LESCs compared to the adjacent cornea and sclera which are stiffer. Several studies have proposed that biomechanical properties, elasticity, and stiffness within the limbal niche may have a role in guiding the proliferation of corneal epithelial cells.^13^

Maturation of the corneal epithelium occurs along a gradient from the limbus towards the central cornea. This is known as XYZ hypothesis, where X is the proliferative phase of the basal epithelium; Y is differentiation and maturation during centripetal migration; and Z is superficial desquamation.^14^ This upward maturation has been demonstrated in the limbal palisades where LESCs are located deeply and transient amplifying cells (TACs) are located more centrally and superficially.^15^

LESCs are progenitor cells with a highly controlled division pattern from the very beginning: one daughter cell remains in the niche to maintain the LESC population while the other one differentiates into a TAC.^16^ The latter is a mitotic cell and increases in number dramatically to post mitotic cells (PMCs) which finally mature into terminally differentiated cells (TDCs), making up the epithelial lining of the cornea.^17^ Recent studies have identified distinct population of LESC/progenitor cells including those that are likely more involved in homeostasis and those that are more actively involved in repair and wound healing.^18,19^ A number of markers have been used to identify the LESC/early progenitor cell population including DeltaNp63,^20^ ABCB5,^21^ ABCG2,^22^ and Keratin 15^23^ while single-cell analyses have proposed more novel makers such as GPHA2,^18,24^ TSPAN7 and SOX17.^24,25^

## Limbal Stem Cell Deficiency (LSCD)

Limbal stem cell deficiency (LCSD) is defined by the absence or impairment of LESCs, leading to the inability to regenerate the corneal epithelium and secondary conjunctival growth over the cornea. There are a number of congenital, traumatic, autoimmune, and exposure-related causes of LCSD.

### Etiology

Deng et al and Vazirani et al have extensively reviewed the underlying causes of LSCD.^26,27^ According to the analysis of globally reported cases, severe chemical injury to the cornea is responsible for at least 75% of all cases requiring LSC transplantation.^27^ LSCD can also be caused by direct damage to the LESCs including thermal injury, multiple surgeries, contact lens wear, and chronic use of benzalkonium chloride -preserved eye drops.^28-31^ Other causes of drug-induced LSCD include topical and systemic medications such as 5-fluorouracil (5-FU), mitomycin-C (MMC), and hydroxyurea.^8^ Immunologically, LESCs can be damaged by various causes such as Stevens-Johnson syndrome, mucous membrane pemphigoid, atopic and vernal keratoconjunctivitis, and graft-versus-host disease.^32-35^ Aniridia is the most common hereditary cause of LSCD. Diabetes may lead to LSCD as evident by a dramatic decrease of putative LESCs marker expression.^36^

### Pathophysiology

Following the loss of limbal stem cells, there is a disruption of the homeostasis of the limbal niche, leading to the clinical manifestations of LCSD.^3^ When a healthy cornea sustains a severe injury, there is an immediate (and delayed) inflammatory response^37^ as well as secretion of cytokines and activation of the LESCs to regenerate the epithelium followed by resolution of the inflammation.^37^ In instances of minor injury, this inflammatory response is appropriately regulated, and the homeostatic state of the limbal niche is restored. However, when the injury is extensive or the limbal niche is compromised, the inflammatory responses are not mitigated, and pathologic changes ensue. Persistent secretion of pro-inflammatory cytokines such as interleukin-1 (IL-1) and its receptor (IL-1R), IL-6, intercellular adhesion molecule-1 (ICAM-1), vascular endothelial growth factor (VEGF), and interferon-ϒ (IFN-ϒ), in addition to defective response of the limbal niche, leads to a vicious cycle that impairs wound healing while further compromising the niche function.^38,39^ It has been shown that colony forming efficiency of LESCs, as well as stem cell markers, are reduced during prolonged inflammation. This unregulated response eventually results in recruitment of epithelial cells from the adjacent conjunctiva.^40^

### Diagnosis of LSCD

**Figure 2.**
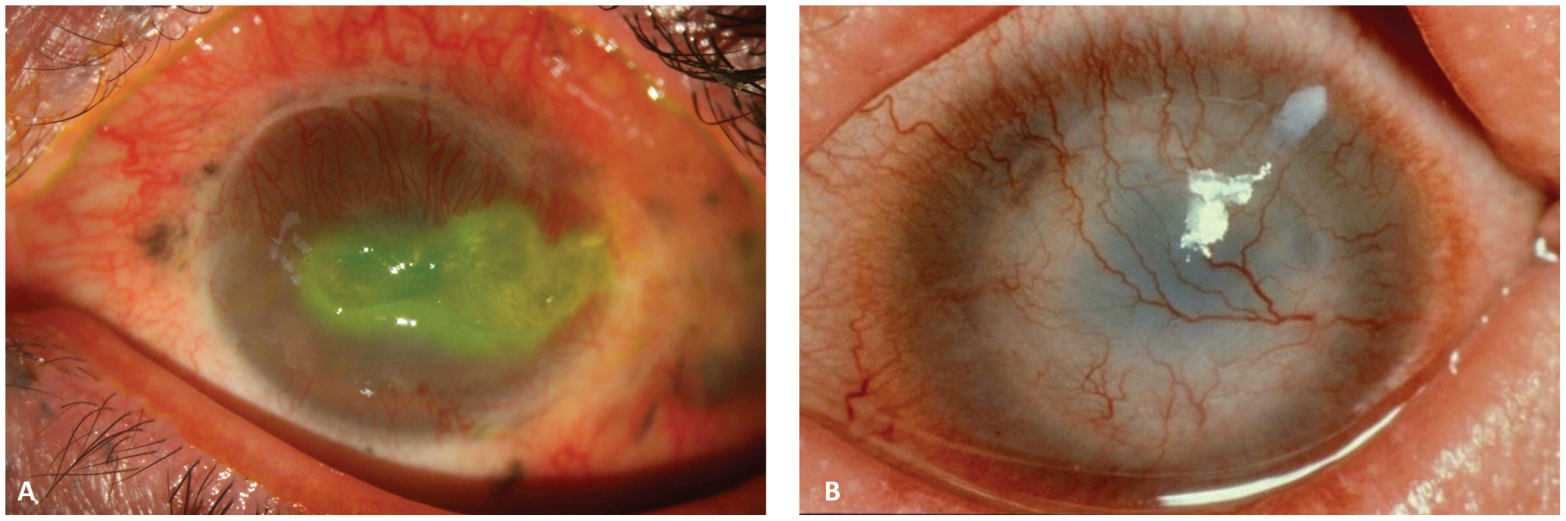
(A) Persistent epithelial defect and corneal vascularization in a patient with a history of explosive chemical injury. (B) Total limbal stem cell deficiency due to genetic disease leading to graft failure and severe corneal vascularization.

Patients suffering from severe LSCD can experience symptoms of decreased vision, pain, photophobia, redness, and tearing.^41^ Sometimes, LSCD may present with debilitating episodes of recurrent corneal erosion, photophobia, and blepharospasm. LSCD can be staged according to the severity on a scale devised by an international working group based on slit-lamp findings (Table 1).^27^ The diagnosis of LSCD is mainly based on slit-lamp examination. Irregular whorl-like fluorescein staining of the cornea, conjunctivalization and neovascularization of the cornea, and loss of limbal anatomy (palisades of Vogt). In later stages, persistent epithelial defects, corneal melting, and perforation can be seen (Fig. 2). Impression cytology is considered the gold standard tool in diagnosing LSCD.^27^ The technique involves the immunohistochemical analysis of the adherent cells obtained by sampling the exposed epithelium on a piece of filter paper made of nitrocellulose acetate, cellulose acetate, or polytetrafluoroethylene. Diagnosis is based on the detection of conjunctival or goblet cell markers in the anatomical corneal regions. This includes cytokeratin 7 and 13 for conjunctival cells, and MUC5AC (or Periodic Acid Schiff staining) for goblet cells.^42,43^

**Table 1.** A staging system for limbal stem cell deficiency based on slit-lamp signs.^27^

Subdivisions	Stage 1: Central 5 mm spared	Stage 2: Central 5 mm affected	Stage 3: Entire cornea affected
A	Less than 50% of limbus affected	Less than 50% of limbus affected	
B	50% or more of the limbus affected	50% or more of the limbus affected (but <100%)	
C	100% of limbus affected		

Developments in anterior segment imaging have led to more accurate diagnosis and staging of LSCD. Table 2 summarizes imaging findings in LSCD using in vivo confocal microscopy (IVCM), anterior segment optical coherence tomography (AS-OCT) and AS-OCT angiography (AS-OCTA). Owing to its cellular level of detail, IVCM is especially useful for staging and monitoring LSCD progression. Reduced subbasal nerve plexus density is also often seen on IVCM. Visualization of goblet cells on IVCM is indicative of late LSCD.^44,47,48^ AS-OCT allows quasi-histological non-contact in vivo imaging of the cornea with 3-dimensional quantification of layer thickness. Central corneal epithelial thickness and limbal epithelial thickness on AS-OCT appear to correlate with LCSD disease, making these measurements a potentially useful diagnostic aid.^44,45^In addition to providing 3-dimensional cross-sectional images of the limbal niche,^44,46^ AS-OCTA provides a volumetric analysis of microvasculature by identifying the change in signal produced by red blood cell motion through sequential scans at the same site.^44^ Recently, it has been reported as a diagnostic tool of LSCD via assessing limbal vessel architecture, limbal vessel density measurements and quantifying extension and depth of corneal vascularization. Increased vascular depth and extensions were found to correlate with LSCD severity.^44^

**Table 2.** IVCM, AS-OCT, and AS-OCTA findings in limbal stem cell deficiency.

IVCM	AS-OCT	AS-OCTA
• Lost normal limbal architecture with poor visualization of the palisades of Vogt; visible goblet cells throughout the conjunctivalised corneal epithelium; blurred epithelial cell contours, sub-basal fibrosis, and reduced sub-basal epithelial cell and nerve plexus density in the central cornea.^44^	• Loss of stromal undulations; loss of normal epithelial thickening in the limbus; loss of clear transition between corneal epithelium and conjunctival epithelium; high corneal epithelial reflectivity; low corneal stromal reflectivity; decreased corneal epithelial thickness.^45,46^	• Increased corneal vascular extension from the limbus to the furthest vessel over the cornea; increased corneal vascular thickness from the most superficial to the deepest corneal vessel.^47^

IVCM: in vivo confocal microscopy, AS-OCT: anterior segment optical coherence tomography, AS-OCTA: anterior segment tomography angiography

## Therapies for LSCD

### Background

Appropriate management of LSCD relies on a combination of medical and surgical approaches.^49^ Graphical abstract summarizes the currently available therapeutic options for LSCD. Medical treatment is considered the mainstay of management for stage 1 LSCD and plays a vital role in stabilizing the disease progression in stages 2 and 3 while the patient is waiting for the definitive surgical intervention. Medical treatments for LSCD include artificial tears, topical or systemic anti-inflammatory medications, topical biologically derived growth factors, topical or systemic drugs that strengthen the corneal structure, and topical or systemic immunomodulatory medications.^50^

When medical therapy fails, or the extent of LSCD is so vast that regeneration is not feasible, surgically transplanted tissue can be used to reconstruct the limbus and restore the stem cell population. The following sections will briefly describe current and emerging techniques for restoring the function of the LESCs with a particular emphasis on restoring the limbal niche.

### Limbal Epithelial Cell Transplants

#### Unilateral LSCD

Limbal epithelial transplantation was first introduced by Barraquer for burn induced LSCD de to improve the epithelization and reduce the inflammation and neovascularization.^31,51^ In late 1980s, Keivyon and Tseng described conjunctival limbal autograft (CLAU) as a treatment for unilateral total LSCD.^52^The procedure includes obtaining 2 grafts of 2 clock hours each from the limbus and the adjacent rim of conjunctiva from the patient’s other healthy eye. This procedure has the longest track record and has been shown to successfully restore the corneal epithelium, in approximately 75% of cases.^53^ Autologous CLAU carries the risk of iatrogenic LSCD in the donor eye as the bed of the graft taken out does not regenerate a normal limbal structure. Eslani et al investigated the long-term outcomes of CLAU in 27 patients with unilateral LSCD with a minimum follow-up of 1 year. They reported that ocular surface stability was achieved in 21 (77.8%) patients while 6 patients (22.2%) developed partial surface failure.^54^

Based on advances in culture techniques, Pellegrini et al described cultivated limbal epithelial transplantation (CLET) for unilateral LSCD in which a small tissue (approximately 2 mm^2^) obtained from the patient’s healthy eye was used to generate corneal epithelial cell sheets in vitro.^55^A carrier such as amniotic membrane or fibrin gel may be used to transplant the cultivated cells onto the recipient bed.^54,56^ Rama et al reported a success rate of 76.6% using CLET in eyes with LCSD from burns; success was associated with a sufficient percentage of limbal stem cells (delta N-p-63 staining) in culture.^57^ On the other hand, Behaegal et al^58^ and Borderie et al^59^ reported a drop in the estimated graft survival from 100% at 3 years to 71% at 5 years that might be attributed to the lack of a healthy LSC niche. Borderie et al demonstrated a statistically difference in the survival rates of the autograft compared to allograft, however there was no difference between the limbal tissue graft and the cultured LESC grafts.^59^ Post-CLET IVCM showed that the original host limbal architecture was not reconstituted.^60^

More recently, simple limbal epithelial transplantation (SLET) was introduced by Sangwan et al as an alternative to CLET with promising mid-term results.^61^ It is technically easier and requires only a 2 × 2 mm (1-clock-hour) limbal block from a healthy contralateral eye. The specimen is then cut into multiple fragments and transplanted over an amniotic membrane with fibrin glue to the recipient cornea.^62,63^ Vazirani et al reported a completely clear cornea in 80% of cases at a median follow-up of 1 year.^64^ They included only LSCD patients with wet ocular surface where chemical and thermal burns were the identified diagnosis in 91.1% of patients.^64^ Basu et al reported a 76% success rate of autologous SLET in patients with ocular chemical burn, at post-operative 1.5 years follow-up.^62^

#### Bilateral LSCD

Allogeneic limbal grafts obtained from cadaver or living donor have been the mainstay of treatment for bilateral total LSCD.^3^ These techniques include keratolimbal allograft (KLAL) and living related-conjunctival limbal allograft (lr-CLAL), respectively. Both KLAL and lr-CLAL necessitate long-term systemic immunosuppression to prevent graft rejection.^3^ An overview of various limbal graft techniques is presented in Table 3.^3,61,65-67^

**Table 3.** Comparison of the surgical options for limbal stem cell deficiency (LSCD).

	Unilateral LSCD	Bilateral LSCD	Systemic immune suppression	Disadvantage	Success rate
**CLAU**	Indicated	NA	Not required	Two pieces of 2-2.5-clock-hour grafts needed; may deplete donor LESCs	80%-100%
**CLET**	Indicated	Allo-CLET	Not required for auto; Required for allo	Expensive; Two-step surgery; Special requirements (feeder cells, special culture systems, a carrier)	70%-77%
**SLET**	Indicated	Allo-SLET	Not required for auto; required for allo	Results less satisfactory when combined with keratoplasty	50%-100%
**KLAL**	NA	Indicated	Always required	Requires cadaveric tissue or living donor Concerns about disease transmission Side effects from systemic immunosuppression Immune-rejection remains a challenge	33%-77%
**CLAL**	NA	Indicated	Always required		

CLAU: conjunctival limbal autograft, CLET: cultivated limbal epithelial transplantation, SLET: simple limbal epithelial transplantation, KLAL: keratolimbal allograft, CLAL: conjunctival limbal allograft, LESCs: limbal epithelial stem cells.

The overall success rate for limbal grafts ranges from 33% to 77% in published studies depending on the cause of LSCD.^3^ Donor cells have been shown to survive long-term when adequate immunosuppression is used. Interestingly, with time host cells also contribute to the epithelium, supporting the hypothesis that rejuvenating the host limbal niche could promote the recipient cells to repopulate the limbal and corneal epithelium.^68^

### Non-limbal Epithelial Cell Transplants

Due to the shortage of allogeneic limbal tissue and the challenges associated with immune rejection of allografts, investigators have studied non-limbal autologous sources of epithelial grafts for bilateral total LSCD.^8^ Cultivated oral mucosal epithelial transplant (COMET) was first described by Nishida et al in 2004.^69^ COMET has been reported to achieve around 43%-67% rate of success in restoring the stability of the ocular surface.^70-72^ However, Kolli et al and Ilmarinen et al reported suboptimal visual outcomes after COMET due to persistent oral mucosal epithelium phenotype with its thicker and less transparent features.^73,74^ Post-COMET peripheral corneal neovascularization has been reported in most patients with at least 83% of examined corneal quadrants showing epithelial neovascularization using AS-OCTA.^75-77^ In the last decade, ex vivo cultivated conjunctival (as opposed to corneal) epithelial autograft was introduced by Ricardo et al with an 86% success rate after 18.5 months of follow up.^78^ Analysis of these conjunctival-based grafts with IVCM revealed well-stratified epithelium with regular hexagonal basal cells.^78^ Although these techniques have proved effective in stabilizing the corneal surface, it appears that oral mucosal and conjunctival epithelial cells lack the optimal characteristics of corneal epithelium.^3^ In addition, limited long-term survival data is available.

As an alternative to COMET and conjunctival grafts, autologous pluripotent stem cells (PSCs) as well as embryonic stem cells (ESCs) have been used to generate corneal epithelial-like cells in an effort to reconstruct the limbal niche. A medium containing limbal fibroblasts on a collagen IV scaffold provided an adequate microenvironment for induction of the ESCs.^79^ Similarly, using microRNA (miRNA)-assisted gene expression, the human induced pluripotent stem cells (hiPSCs) were successfully differentiated into corneal epithelium-like cells.^80^ Finally, corneal epithelial stem cells and progenitor cells isolated in the presence of rho-kinase inhibitor and keratinocyte growth factor form a self-formed ectodermal autonomous multi-zone (SEAM) tissue (mimicking whole eye development) which was used isolate the corneal epithelium and restore the corneal surface in animal models.^81,82^ While early phase clinical trials of this latter technique are underway, safety and cost considerations will need to be addressed before these methods can be widely adopted in the clinic.^83^

### Mesenchymal Stromal Stem Cells

MSCs are multipotent cells that can be isolated from different tissues such as bone marrow, fat, and corneal-limbal stroma.^84^ MSCs have been shown to produce extracellular matrix in 3 dimensional culture systems and affect innate and acquired immune responses by secreting anti-inflammatory and growth factors.^85^

MSCs are especially attractive as a potential therapeutic option since they may lead to restoration of a defective niche, which is invaluable for the maintenance and longevity of epithelial transplants.^3^ MSCs derived from limbal tissue were found to decrease corneal opacification and neovascularization in rat alkali-burn models via topical or subconjunctival routes.^86^ Furthermore, the secretomes (supernatant) of the in vitro cultivated limbal MSCs have been reported to promote corneal epithelial regeneration while suppressing inflammation and neovascularization.^87,88^ A study by Shibata et al showed that the secretomes of adipose derived MSCs can suppress epithelial-mesenchymal transition in human corneal epithelium.^89^ Eslani et al evaluated the angiogenic properties of corneal derived MSCs and reported that corneal derived MSCs secrete high levels of antiangiogenic factors (pigment epithelial growth factor and soluble fms-like tyrosine kinase-1) and low levels of VEGF-A. Those factors resulted in significant reduction of new vessel formation.^88^ MSCs secrete cytokines such as epidermal growth factor (EGF) and immunomodulatory proteins such as tumor necrosis factor stimulated gene/protein-6 (TSG-6).^90^ In vitro animal studies have shown that bone marrow MSCs co-cultured with LESCs transdifferentiate into cells with corneal epithelial markers.^91^ A similar finding was observed using human adipose MSCs.^92^ Despite the established beneficial role of MSCs, the available clinical data is still limited.^93,94^ The first clinical trial using MSCs, conducted by Calonge et al comparing allogeneic bone marrow MSC transplantation with CLET, revealed similar safety and efficacy profiles between the 2 methods after 12-month follow-up.^95^ Likewise, early studies have reported acceptable clinical safety following local administration in patients with severe dry eye disease and acute chemical injuries.^96,97^

## Bioengineered Extracellular Matrix

ECM plays a vital role in maintaining the limbal stem cell niche and supports limbal epithelial stem cell functions. Amniotic membrane, a widely used ECM scaffold for ocular surface reconstruction, has a collagen- and laminin-rich basement membrane,^98^ which promotes epithelial cell migration, adhesion, proliferation, and differentiation.^99^ With its anti-inflammatory, antiangiogenic, and antifibrotic properties, amniotic membrane has been a useful tool in tissue engineering and cell delivery.^99,100^ However, it does have some drawbacks. It is less transparent and digests shortly after transplantation, rendering its role only temporarily effective.^99^ Other novel ECM substitutes that have been proposed as carriers for cultivated limbal epithelial cells include ECM hydrogels, collagen, fibrin, siloxane hydrogel contact lenses, and silk fibroin.^101-104^ Likewise, ECM hydrogels have been produced from decellularized porcine corneal stroma or through 3 dimensional bioprinting technology via mixing collagen, elastin, and laminin as bio-inks.^105^ Recently, Yazdanpanah et al reported the potential regenerative effects of an ocular bandage hydrogel made from decellularized porcine corneal ECM on a murine corneal epithelial wound healing model.^106^ Using soft lithography, silk fibroin can be prepared as highly translucent films and altered to create nanoscale models to imitate the ECM structures.^107^ These biomaterials are potential platforms for transplantable tissue-engineered corneal epithelial cell sheets or stem cell niche and may offer promising options for patients with limbal stem cell deficiency.^108^

## Growth Factors to Revitalize the Limbal Niche

As mentioned before, the limbal niche homeostasis is highly dependent on the proper signaling and crosstalk between its cellular components. Local administration of exogenous growth factors is considered a non-invasive approach to help restore the limbal niche function.^109^

Being rich in EGF, Transforming Growth Factor-beta (TGF-β), fibronectin, vitamin A and many other cytokines, autologous serum eye drops have been found to restore a healthier ocular surface in patients with graft-versus-host disease, dry eye disease, Sjögren’s disease and LSCD.^110^ Likewise, platelet-derived preparations, such as platelet releasate (PR),^111^ plasma rich in growth factors (PRGF),^112^ and platelet-rich plasma (PRP),^109^ were found to have growth factors (eg, EGF, TGF, pigment-epithelium derived factor (PEDF), basic fibroblast growth factor (bFGF), and insulin-like growth factor-1 (IGF-1)) essential for regenerating the limbal niche.

Baradaran-Rafii et al reported the regenerative role of amniotic membrane extract eye drops (AMEED) in the setting of in vivo cultivation of limbal stem cells in patients with LSCD.^113^ Rugg et al^114^ described a growth factor (HC1-HA/PTX3), purified from AM, that was found to promote the self-renewal of the LESCs by modulating the Wnt/BMP signaling in 3-dimensional culture systems.^115^

Self-renewal of LESCs was also found to be promoted via PEDF.^116^ Ho et al reported that PEDF was found to activate mitogen-activated protein kinase (MAPK) and signal transducer and activator of transcription (STAT3) pathways and subsequently enhanced corneal epithelial healing.^116^

Despite the reported beneficial roles of the aforementioned growth factors, their efficacy in severe epithelial disease is still limited.^3^ Further investigations are required to provide more effective growth factor cocktails as a potential topical therapeutic option for LSCD.

## Future Trends

Surgical interventions for LSCD are categorized by the source of stem cells (eg, autologous, allogeneic) and type of stem cell graft (eg, limbal, non-limbal) and the use of ex vivo expansion of stem cells in culture. It is now clear that any therapeutic approaches comprising of stem cells will fail if the appropriate stem cell niche is not restored.^117^ Future therapies should address the need for improved survival and function of the grafts, possibly through the administration of topical growth factors or using novel biomaterial platforms. Creation of HLA-matched iPSCs and reprogrammed differentiated cell lineages (instead of inducing PSCs) are forthcoming interests.^118^ Application of MSCs, and other alternative stem cell sources, into clinical practice is an active field of research with clinical trials underway.^84^ Topical preparations such as secretomes derived from ex vivo cultures of MSC contain many growth factors that enhance the viability or regenerative capacity of limbal stem cell niche.^84,87^

## Conclusion

LSCD is a rare and potentially blinding disease of the cornea. Treatment options depend on the extent (partial or total; unilateral or bilateral) and severity of LSCD. Although, effective therapeutic approaches to replenish the corneal epithelium have been developed over the past 3 decades, there is still an unmet need.

For unilateral LSCD autografts including CLAU, CLET, and SLET have been successful in restoring the corneal phenotype in great majority of patients. Bilateral LSCD with extensive damage is more challenging and requires allograft transplantation from cadaver (KLAL) or living donor (CLAL) along with long-term systemic immunosuppression.

MSCs and stem cells from allogeneic or autologous non-limbal sources with their immunomodulatory characteristics and the ability to support epithelial cells have been studied with promising results.

## Data Availability

Data sharing is not applicable to this article as no new data were created or analyzed in this study.
